# The WD40 gene family in recretohalophyte *Limonium bicolor*: genomic identification and functional analysis in salt gland development and salinity tolerance

**DOI:** 10.3389/fpls.2025.1629604

**Published:** 2025-07-30

**Authors:** Lu Sun, Huiying Mu, Yuqing Tan, Baoshan Wang, Xi Wang, Fang Yuan

**Affiliations:** ^1^ Shandong Provincial Key Laboratory of Plant Stress, College of Life Sciences, Shandong Normal University, Jinan, Shandong, China; ^2^ Shandong Provincial Key Laboratory for Biology of Vegetable Diseases and Insect Pests, College of Plant Protection, Shandong Agricultural University, Taian, China; ^3^ National Center of Technology Innovation for Comprehensive Utilization of Saline-Alkali Land, Dongying, Shandong, China

**Keywords:** feature description, Limonium bicolor, subcellular localization, salt gland development, salt tolerance, WD40 gene family

## Abstract

**Introduction:**

Developing salt-tolerant crops is critical for utilizing saline soils in agriculture. *Limonium bicolor*, a recretohalophyte with epidermal salt glands, represents a valuable genetic resource for salt tolerance engineering. Although WD40 proteins are known regulators of plant stress responses, their roles in *L. bicolor* remain unexplored.

**Methods:**

We performed a genome-wide analysis of WD40 genes in *L. bicolor*, including phylogenetic classification, subcellular localization prediction, cis-element analysis, and expression profiling during salt stress. Functional validation was conducted using virus-induced gene silencing (VIGS).

**Results:**

Among 367 identified WD40 genes (distributed across all chromosomes), Subfamily 6 was the largest. Two key members (Lb1G05968 and Lb3G17197, localized in cytoplasm) showed significant involvement in salt gland development and stress tolerance, as demonstrated by VIGS-induced phenotypic defects.

**Discussion:**

Our findings reveal the WD40 family's expansion in *L. bicolor* and its functional specialization in salt adaptation. The identified genes (e.g., Lb1G05968, Lb3G17197) provide targets for engineering salt-tolerant crops. This study establishes a foundation for further research on halophyte developmental genetics.

## Highlights

- We have identified and explored the predicted properties of the WD40 protein family in the sea lavender *Limonium bicolor*, consisting of 367 members. We demonstrated the participation of two members in salt gland development and salt tolerance.

## Introduction

Saline-alkali land is globally distributed, affecting over 100 countries and currently covering a total area of 1.38 billion hectares (Zhou et al., 2023), accounting for about 10.7% of the world land surface (Lekka et al., 2023). As the human population is projected to reach 9.5 billion over the next 50 years, the efficient utilization of saline-alkali land is becoming an urgent global challenge to enhance crop productivity (Fu et al., 2021). Current traditional strategies for the valorization of saline-alkali soil include chemical methods like applying gypsum or phosphogypsum, and physical methods such as deep plowing to suppress salt accumulation in the topsoil (Gu et al., 2023). However, traditional methods often disrupt ecological balance and incur high economic costs (Castro-Díez et al., 2019; Zhang et al., 2023). By contrast, improving the salinity tolerance of crop species to utilize saline-alkali land represents a more economical and innovative strategy (Zhou et al., 2022).

Halophytes, which can complete their life cycles in high-salinity environments, serve as valuable research models to enhance our understanding of mechanisms behind salinity tolerance (Bueno and Cordovilla, 2019). Traditionally, halophytes are classified into three categories: euhalophytes, pseudo-halophytes, and recretohalophytes (Yuan et al., 2018a). The sea lavender *Limonium bicolor*, a recretohalophyte, possesses specialized salt glands that can excrete excessive salts. Each salt gland is a 16-cell multicellular complex composed of four secretory cells, four subsidiary cells, four inner cup cells, and four outer cup cells. Ultrastructural observations have revealed abundant mitochondria within salt gland cells and dense plasmodesmata connecting salt gland cells and their neighboring mesophyll cells (Yuan et al., 2015a). A systematic exploration of salinity tolerance–related genes in *L. bicolor* will elucidate the molecular mechanisms underlying halophyte adaptation to salinity and lay the foundation for transferring salinity tolerance genes to crops through genetic engineering.

WD40 proteins, also known as WD repeat (WDR) or WD40 repeat proteins, carry a highly conserved WD40 motif. The central role of this domain is to orchestrate protein-protein interactions, underpinning its significance in numerous biological pathways. This motif starts with a glycine-histidine (GH) pair at its N terminus and ends with a tryptophan-aspartic acid (WD) pair at its C terminus, also known as the Trp-Asp motif (Neer et al., 1994; Awdhesh et al., 2012). The name WD40 derives from the conserved WD residues and is given to a core domain of approximately 40 amino acids per repeat (Smith et al., 1999). WD40 proteins typically contain 4–16 tandem repeating WD motifs, each consisting of four antiparallel beta-folded chains (Xu and Min, 2011). About 1–2% of all proteins in eukaryotes harbor WD40 domains, whereas this domain is rarely found in prokaryotes (Stirnimann et al., 2010). The WD40 superfamily has been described in Arabidopsis (*Arabidopsis thaliana*) (Li et al., 2014), cucumber (*Cucumis sativus*) (Yang et al., 2018), foxtail millet (*Setaria italica*) (Mishra et al., 2014), and rice (*Oryza sativa*) (De Vetten et al., 1997).

Within cells, WD40 proteins localize to the cytoplasm or nucleus: they are attached to the cytoskeleton or membranes by binding to membrane proteins or to the membrane via helper domains (Liu et al., 2023a). Known WD40 proteins range in size from small proteins, such as the 32-kDa PLEIOTROPIC REGULATORY LOCUS 1 (PRL1), which is involved in sugar signaling transduction and stress responses (Meng et al., 2024), to large proteins (> 400 kDa), such as the mammalian lysosomal trafficking regulator Lyst (Turner et al., 2024). WD40 proteins are closely associated with a variety of cellular and biological programs, including cell division, apoptosis, light signaling and vision, cell movement, flowering, flower development, and cell differentiation (Sompornpailin et al., 2002; Simlat et al., 2017). One of the most important functions of WD40 proteins in plants is their involvement in anthocyanin biosynthesis and regulation (Carey et al., 2004; Yao et al., 2017). The other most reported functions are focused on the responses to stresses. In rice, the OsABT gene encodes a protein containing seven WD40 domains, and promotes salt tolerance by heterologous expression in *Arabidopsis* (Eryong and Bo, 2022). Similar reports are seen in legume that a conserved legume-specific WD40/YVTN protein was identified to play a positive regulatory role in NaCl response (Haluk et al., 2023). In *Chlamydomonas reinhardtii*, HpXBCP3 enhances microalgal sensitivity to NaCl stress by regulating the expression of ubiquitin and WD40 proteins (Liu et al., 2021).

While halophytes offer large resources for genes contributing to salinity tolerance, limited studies have documented WD40 proteins in *L. bicolor*. The WD40 protein LbTTG1 (encoded by Lb1G04050) negatively regulated salt gland development and salinity tolerance (Yuan et al., 2022) by modulating the development of epidermal structures and response to stress in *L. bicolor* (Yuan et al., 2019). To expand our analysis, we systematically identified all members of the WD40 gene family in *L. bicolor*, analyzed their chromosomal position, and tested their function in the development and stress responses of *L. bicolor*. Based on their expression patterns under NaCl treatment, we characterized two *LbWD40* genes with potential roles in the growth and stress tolerance of *L. bicolor*. This study provides insights into the salt tolerance mechanism of *L. bicolor* and establishes a foundation for improving salt tolerance in crop plants.

## Results

### Identification of the WD40 gene family in *Limonium bicolor*

We identified 367 WD40 family genes in the *L. bicolor* genome and anchored them to one of the eight *L. bicolor* chromosomes. We confirmed the presence of at least one WD40 domain in each protein using the Pfam_scan program. The nucleotide lengths of the genomic sequences for *LbWD40* genes ranged from 357 bp to 13,401 bp, with an average of 6,879 bp (Zhang et al., 2024). Among them, Lb2G14320 encoded the shortest protein (119 amino acids, aa), while Lb5G28947 encoded the longest protein (4,467 aa), with an average protein length of 2,293 aa. The molecular weights (MW) of putative LbWD40 proteins ranged from 12.63 kDa (encoded by Lb2G14320) to 496.82 kDa (encoded by Lb5G28947), with an average MW of 254.72 kDa. The isoelectric points (pI) of these proteins varied between 4.17 (for the proteins encoded by Lb4G26257 or Lb4G26257) and 10.18 (for the protein encoded by Lb7G33999), averaging 7.175 (**Supplementary Table S1**). Notably, the nucleotide lengths, protein lengths, molecular weights, and pI values of these WD40 genes covered an order of magnitude in their range. These findings highlight the comprehensive identification of *LbWD40* genes in this study, laying the groundwork for a systematic functional analysis of this gene family in *L. bicolor*.

### Phylogenetic analysis of WD40 genes in *L. bicolor* and Arabidopsis

**Figure 1:**
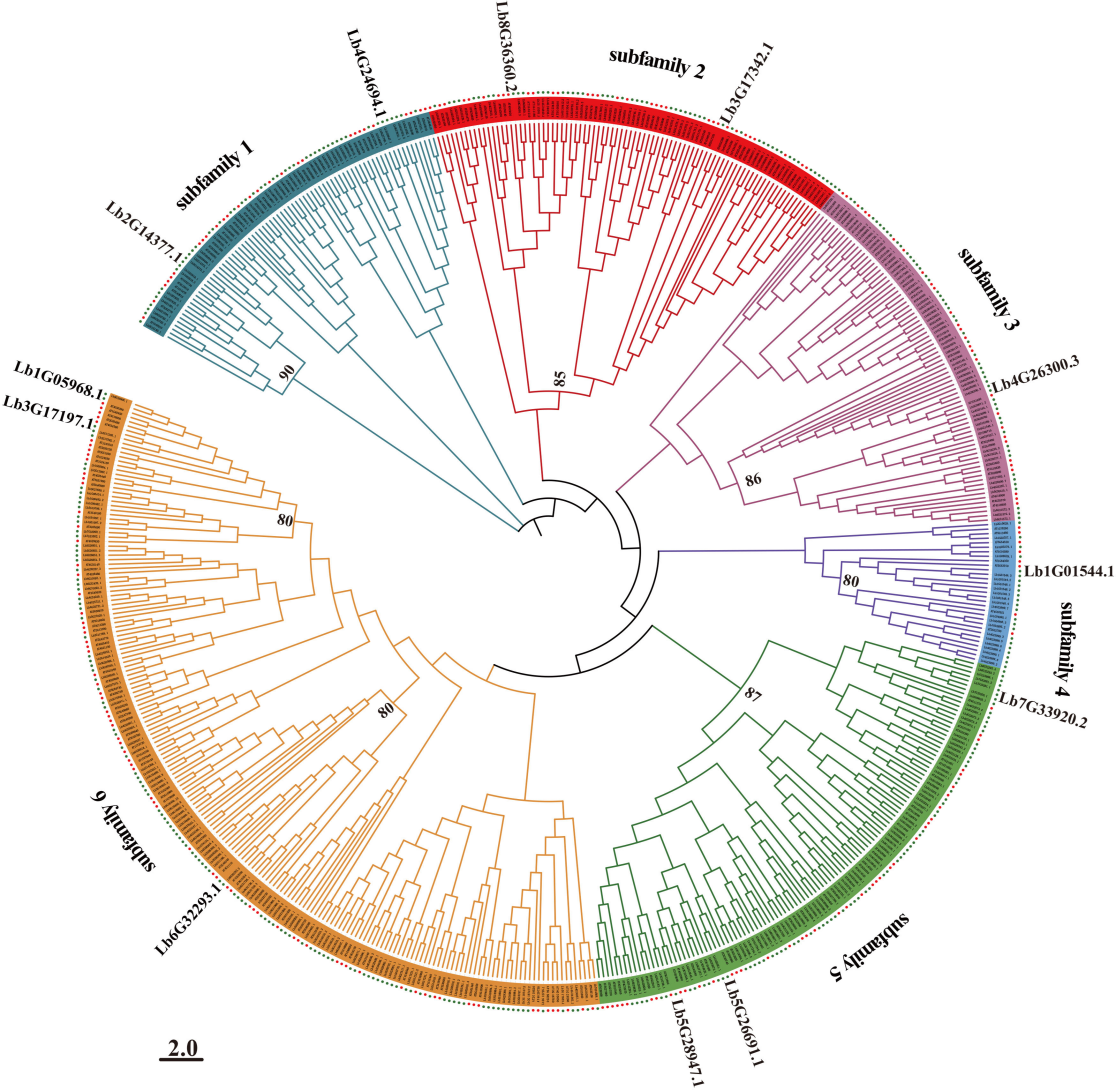
Phylogenetic tree of the WD40 family members from *Limonium bicolor* and Arabidopsis. A multiple amino acid sequence alignment was obtained with MUSCLE (employing gap penalties of -400 for opening and 0 for extension, with UPGMB clustering); The phylogenetic tree was reconstructed based on the maximum-likelihood method in MEGA 7.0. The scale bar indicates number of amino acid substitutions per site. Bootstrap values of significant branches within the six subfamilies (>50%) from 1,000 replicates are shown at key nodes. The red dots represent WD40 family members from *Limonium bicolor*, while the green dots denote WD40 family members from *Arabidopsis thaliana*.

We performed a multiple sequence alignment of the 213 WD40 proteins from Arabidopsis and the 367 WD40 proteins identified in *L. bicolor*. We then used the alignment as input for MEGA to reconstruct a maximum-likelihood phylogenetic tree (Wang et al., 2024), which we visualized with the iTOL program (**Figure 1**). Based on evolutionary relationships and the numbers of WD40 repetition, we classified the WD40 genes into six major subfamilies, with members within each subfamily originating from the same phylogenetic branch. Each subfamily contained members from both Arabidopsis and L. bicolor. Subfamily 6 had the most *L. bicolor* members, with 118 WD40 proteins. The second-largest subfamily was subfamily 5, with 73 WD40 proteins. Subfamily 4 had the fewest WD40 proteins, with only 23 members.

### Analysis of gene structure and sequence composition

To investigate the evolutionary history of the WD40 gene family, we systematically analyzed the number and arrangement of exons and introns by comparing the coding sequences to their corresponding genomic sequences, revealing the diversity of gene structures. The 367 *LbWD40* genes encoded multiple DNA-binding domains and exhibited highly variable exon numbers, ranging from 1 (Lb1G03818) to 51 (Lb0G37653) (**Supplementary Figure S1**). Notably, Lb7G33920 was derived from the longest gene model, which contained 23 introns, while Lb3G20230 had a short sequence with no intron. Generally, *LbWD40* genes within the same phylogenetic clade shared similar exon numbers. *LbWD40* genes from the same subfamily also exhibited comparable exon counts and gene structures, suggesting conserved evolutionary patterns.

We identified 38 possible functional domains within LbWD40 proteins by conducting a MEME analysis (**Supplementary Figure S2**). All proteins contained the WD40 motif, with 248 proteins (67.5% of total) exclusively harboring only this motif. The proteins encoded by Lb5G28947 and Lb1G01720 each contained five distinct functional motifs, the most of all proteins analyzed. Furthermore, most WD40 proteins within the same subfamily shared the same motif configuration, reinforcing the structural and functional coherence of subfamilies.

### Analysis of chromosome distribution, tandem gene duplication, and segmental gene duplication of *LbWD40* genes

**Figure 2:**
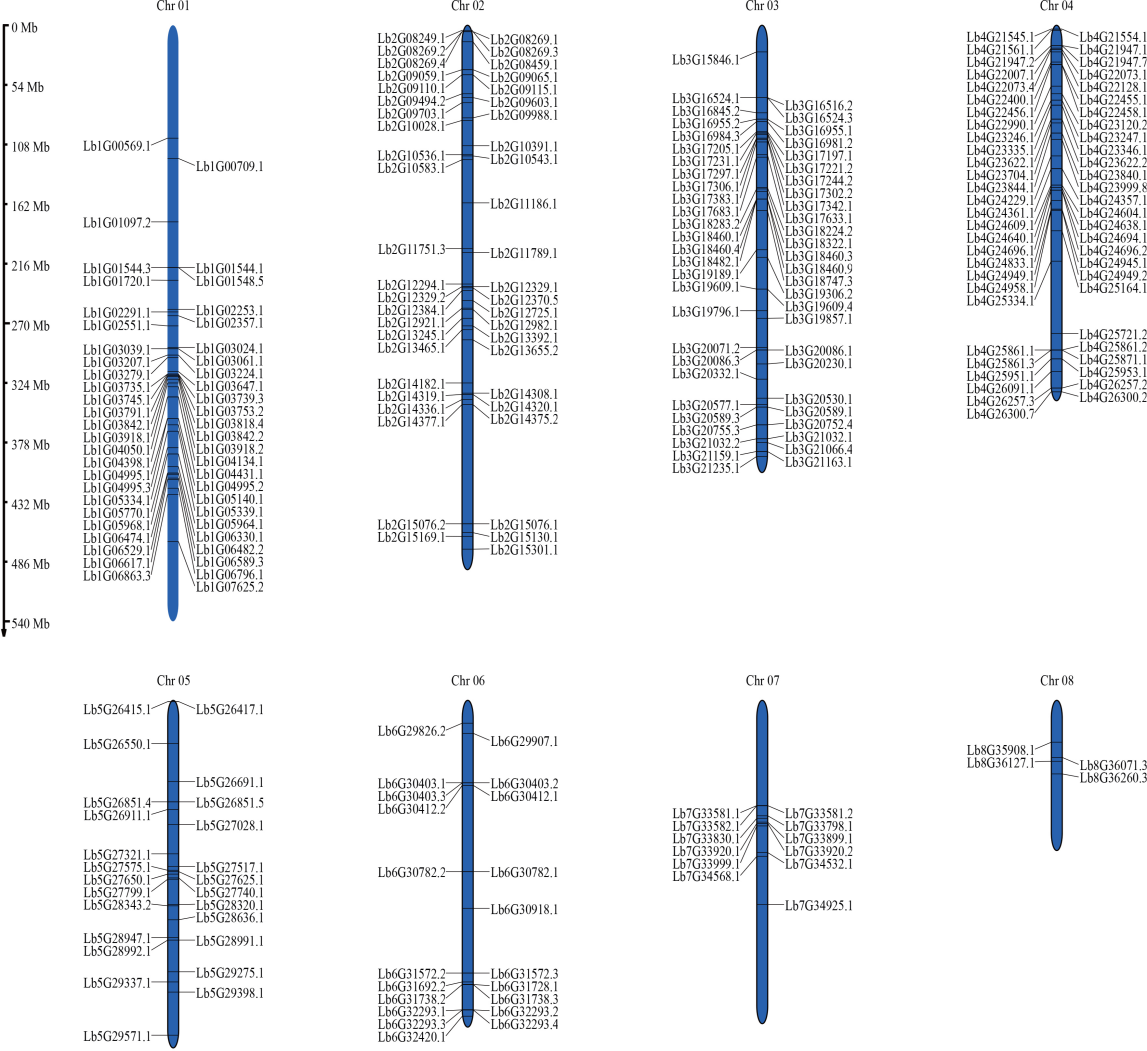
Chromosomal distribution of *LbWD40* gene family members in *Limonium bicolor*. Chromosomal localization analysis was performed for 367 LbWD40 gene family members. The chromosome numbers are indicated at the top of each corresponding chromosome. Chromosome lengths in the figure are measured in megabases (Mb).

**Figure 3:**
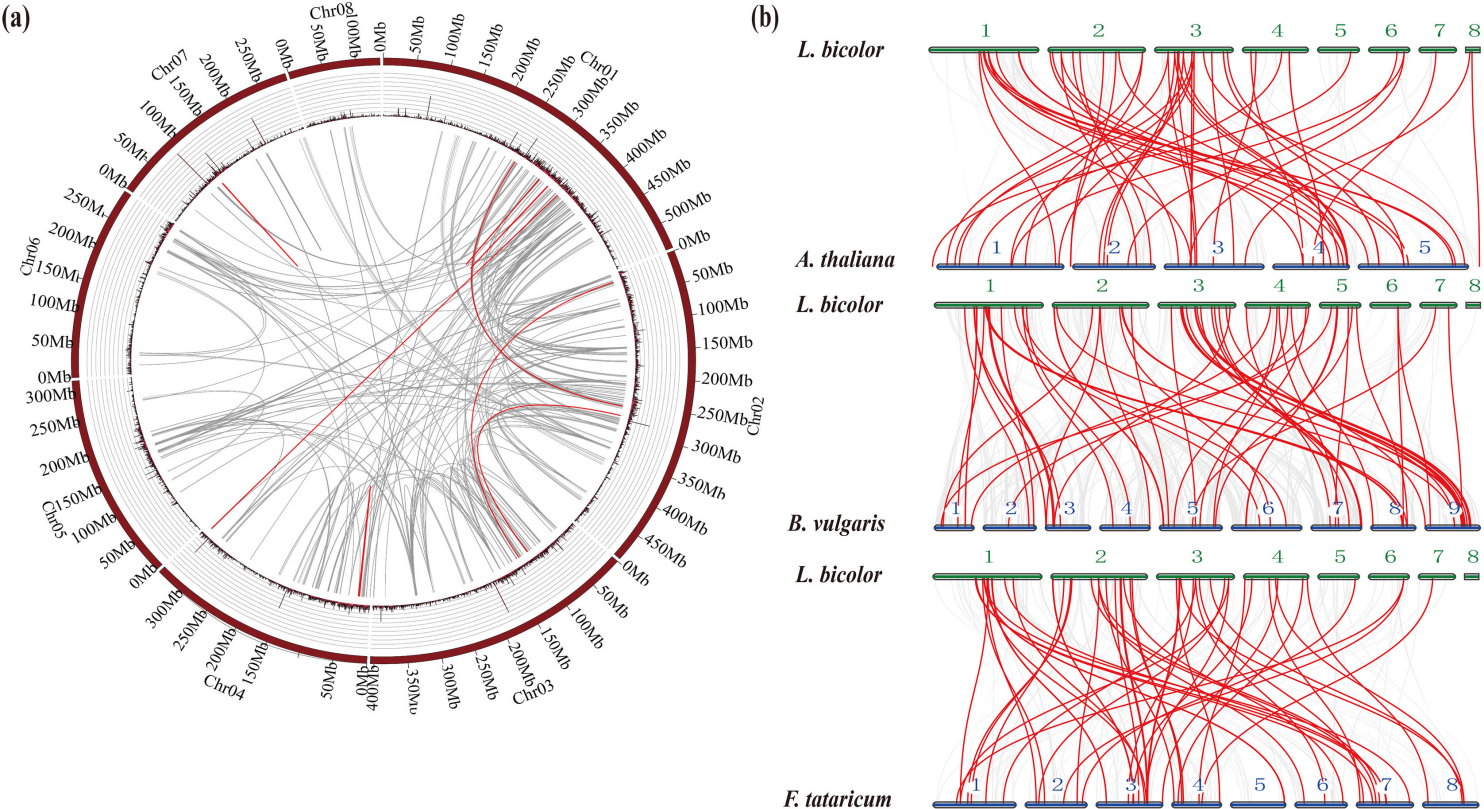
Chromosomal relationship and homology analysis of the *Limonium bicolor* WD40 gene family. **(a)** Chromosomal relationship of WD40 genes in *L. bicolor.* The gray lines represent colinear blocks in the genomes of *L. bicolor*, while the red lines emphasize colinear *LbWD40* gene pairs. **(b)** Homology analysis of the WD40 gene family of *L. bicolor* and three representative plant species: *Arabidopsis thaliana*, sugarbeet (*Beta vulgaris*), and Tartary buckwheat (*Fagopyrum tataricum*).

The 367 WD40 genes were unevenly distributed along all eight chromosomes of the *L. bicolor* genome (**Figure 2**). There were 68, 55, 86, and 76 genes on chromosome 1, chromosome 2, chromosome 3, and chromosome 4, respectively. Chromosomes 5, 6, and 7 had fewer genes, with 29, 25, and 12, respectively. Chromosome 8 had the smallest number of genes, at just eight. We identified 8 pairs of tandemly duplicated genes, which were unevenly distributed on 8 chromosomes (**Figure 3a**). There was one pair of duplicated genes (Lb1G01544 and Lb1G01548) on chromosome 1, two pairs on chromosome 2 (Lb2G08269 and Lb2G08269, Lb2G08269 and Lb2G08269), five pairs on chromosome 3 (Lb3G16516 and Lb3G16524, Lb3G17297 and Lb3G17306, Lb3G17302 and Lb3G17342, Lb3G184603 and Lb3G184609, Lb3G18460 and Lb3G184604), and 3 pairs on chromosome 6 (Lb6G30403 and Lb6G30403, Lb6G32293 and Lb6G32293, Lb6G32293 and Lb7G33588 with Lb7G33582).

In addition to the tandem duplications, we detected 11 pairs of segmental duplications in the *L. bicolor* WD40 gene family (**Figure 3a**). In summary, several *LbWD40* genes were generated by tandem duplication or segmental duplication, which may be one of the driving factors of *LbWD40* gene evolution (Cannon et al., 2004).

### Evolutionary analysis of the *LbWD40* gene family and WD40 genes in different plant species

In addition to the WD40 genes from Arabidopsis and *L. bicolor*, we obtained the sequences for all WD40 genes in two dicotyledonous plant species: sugar beet (*Beta vulgaris*) and Tartary buckwheat (*Fagopyrum tataricum*). We chose *B. vulgaris* and *F. tataricum* because they share a high degree of phylogenetic and evolutionary similarity with *L. bicolor* (Yuan et al., 2022), while Arabidopsis is an important model system. We used the JCVI to extract colinear blocks between *L. bicolor* and the genomes of Arabidopsis, *B. vulgaris*, and *F. tataricum*. We then extracted the WD40 genes mapping to within these colinear blocks to conduct a synteny analysis.

The WD40 genes in Arabidopsis, *B. vulgaris*, and *F. tataricum* were distributed across all chromosomes of their respective genomes. The synteny analysis showed that the WD40 genes of *L. bicolor* form homologous pairs with genes from the other plant species. Among them, Arabidopsis had the highest number of homologous pairs with *LbWD40* genes (48 pairs), followed by *B. vulgaris* with 47 pairs, and *F. tataricum* with 42 pairs (**Figure 3b**).

### Analysis of *LbWD40* expression levels

To explore the potential contribution of *LbWD40* genes to the development of salt glands in *L. bicolor*, the developmental transcriptome was enriched from the first true leaf at each developmental stage (A-E stage) representing distinct phases of salt gland development, which could be divided into five stages: Stage A (undifferentiated phase with no salt gland initiation), Stage B (salt gland differentiation phase), Stage C (stomatal differentiation phase), Stage D (epidermal cell differentiation phase), and Stage E (maturation phase). Using these expression data, we performed a hierarchical clustering heatmap with the “pheatmap” package in R (**Supplementary Figure S3**). Most *LbWD40* genes were highly expressed during early salt gland development (Stages A and B), suggesting their possible involvement in the initiation of salt glands.

To analyze the temporal dynamics of *LbWD40* gene expression under salinity stress, we examined transcriptome data from seedlings treated with 200 mM NaCl at five time points (0, 12, 24, 48, and 72 h) (**Supplementary Figure S4**). Hierarchical clustering of the data and visualization as a heatmap revealed two distinct expression patterns: most *LbWD40* genes were highly expressed within 12 h of NaCl treatment, while a smaller subset was highly expressed at 24-72 h after NaCl treatment. The heterogeneous expression profiles observed among the 367 WD40 genes suggest differential responsiveness to NaCl treatment.

### Silencing of two *LbWD40* members confirms their involvement in salt gland development and salt secretion in *L. bicolor*

**Figure 4:**
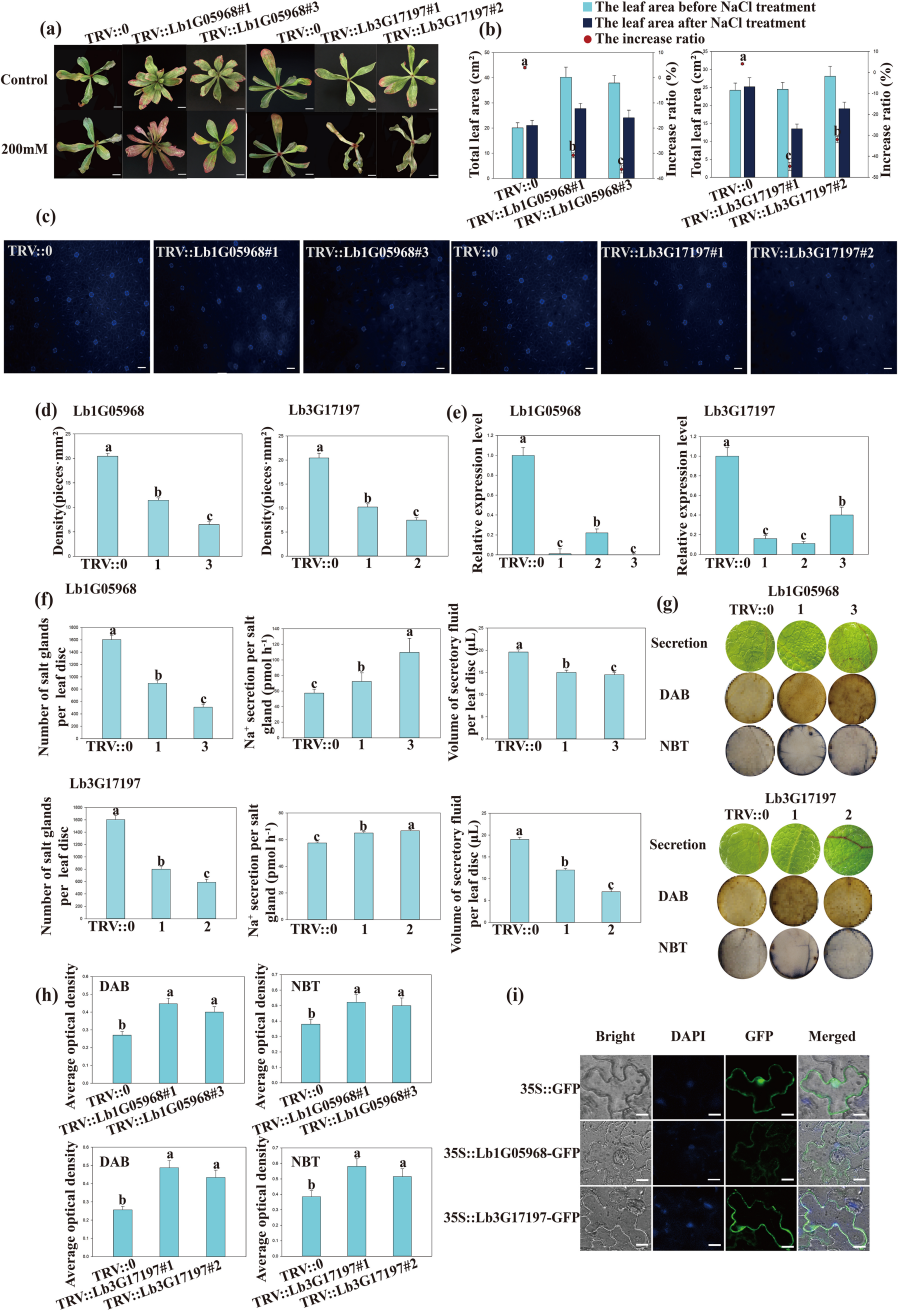
Exploring the roles of Lb1G05968 and Lb3G17197 based on expression patterns and silencing in *Limonium bicolor* leaves. SPSS was used to determine the statistical significance of the data. Different letters indicate significant differences (p = 0.05; Duncan’s multiple range test). **(a)** The Phenotype images of wild type and mutant under salt stress. Scale bars, 2 cm. **(b)** The total leaf area changes and increase ratio (%) before and after NaCl treatment in the TRV:: 0 control and Lb1G05968 and Lb3G17197 silence lines. **(c)** Representative photographs of salt glands on the epidermis of leaves from L. bicolor plants infiltrated with the control pTRV::0 vector or the silencing constructs for Lb1G05968 (pTRV::Lb1G05968) or Lb3G17197 (pTRV::Lb3G17197). Two independent plants are shown for each VIGS (Virus-Induced Gene Silencing construct): TRV::Lb1G05968 #1, TRV::Lb1G05968 #3, TRV::Lb3G1719 #1, and TRV::Lb3G17197 #2. Scale bars, 50 μm. **(d)** Salt gland density in the leaf epidermis of plants infiltrated with pTRV::0, pTRV::Lb1G05968, or pTRV::Lb3G17197. Two independent plants are shown for each VIGS construct: TRV::Lb1G05968 #1, TRV::Lb1G05968 #3 and TRV::Lb3G17197 #1, TRV::Lb3G17197 #3. **(e)** Relative expression levels of Lb1G05968 and Lb3G17197 in TRV::0 control plants and TRV::Lb1G05968 or TRV::Lb3G17197 plants, as determined by RT-qPCR. **(f)** Salt secretion indicators for the leaves of TRV::0 and TRV::Lb1G05968 or TRV::Lb3G17197 plants: number of salt glands, concentration of secreted Na+, and volume of secreted fluid. **(g)** Assessment of salt secretion by the leaf disc method. Representative images of leaf discs show the salt secretion fluid collecting on the disc surface. Leaf disc damage under salt treatment was estimated by DAB and NBT staining in leaf discs from control (TRV::0) and LbWD40-silenced leaves (TRV::Lb1G05968 #1, TRV::Lb1G05968 #3 and TRV::Lb3G17197 #1, TRV::Lb3G17197 #2). **(h)** Quantitative analysis of staining degree based on average optical density data measured by ImageJ. **(i)** Localization of Lb1G05968 and Lb3G17197. Each coding sequence was cloned in-frame and upstream of GFP (Green Fluorescent Protein); the resulting construct was infiltrated into Nicotiana benthamiana leaves. Scale bars, 10 μm. Nuclei were stained with DAPI.

Given that subfamily six contains the largest number among all subfamilies, we chose two LbWD40 genes (Lb1G05968 and Lb3G17197, with high homologies (**Supplementary Figure S5**) with REBC of *Chenopodium quinoa*) for functional characterization. Using virus-induced gene silencing (VIGS), we produced plants silenced for either gene following *Agrobacterium tumefaciens*-mediated infiltration of the respective construct into the leaves of *L. bicolor* plants. As control, we used the empty vector pTRV::0. After 200 mM NaCl treatment, We determined that the silenced lines exhibited less leaf expansion compared to the TRV::0 group following salt treatment (**Figures 4a, b**). We examined salt glands on the leaf surface, using their autofluorescence property under illumination with ultraviolet-B (UV-B) light. The silenced lines have fewer salt glands than the TRV::0 controls, with up to a 66% drop in salt gland number per field of view (**Figures 4c, d**) after identifying the Lb1G05968 and Lb3G17197 expression (**Figure 4e**).

We turned to leaf disc assays to assess the salt secretion from control and silenced *L. bicolor* leaves. While the total salt secretion per unit leaf area was lower in the silenced plants than in the TRV::0 control, the secretion rate per salt gland rose (**Figure 4f**). Considering that lower salinity tolerance might cause cellular damage, we employed diaminobenzidine (DAB) and nitroblue tetrazolium (NBT) histochemical staining to assess the degree of reactive oxygen species (ROS) accumulation under salt stress. The intensity of leaf staining was greater in the silenced plants for each gene than in the control group (**Figures 4g, h**). We therefore conclude that Lb1G05968 and Lb3G17197 positively regulate salt gland differentiation and total salt secretion.

### Subcellular localization of two LbWD40 proteins

We generated constructs encoding a fusion between the green fluorescent protein (GFP) and the protein encoded by Lb1G05968 or Lb3G17197 into a modified pCAMBIA1300 vector. We introduced these constructs individually into the epidermal cells of *Nicotiana benthamiana* leaves. After 36 h of cultivation post infiltration, we examined GFP fluorescence signals by confocal laser scanning microscopy. We detected GFP fluorescence at the plasma membrane for both constructs, while we observed GFP signals from free GFP at the plasma membrane and nucleus (**Figure 4i**). We hypothesize that these two LbWD40 proteins may be associated with the cytoskeleton or membrane, possibly with a role in the early development of epidermal structures.

### Two LbWD40 family members interact with LbHLH

**Figure 5:**
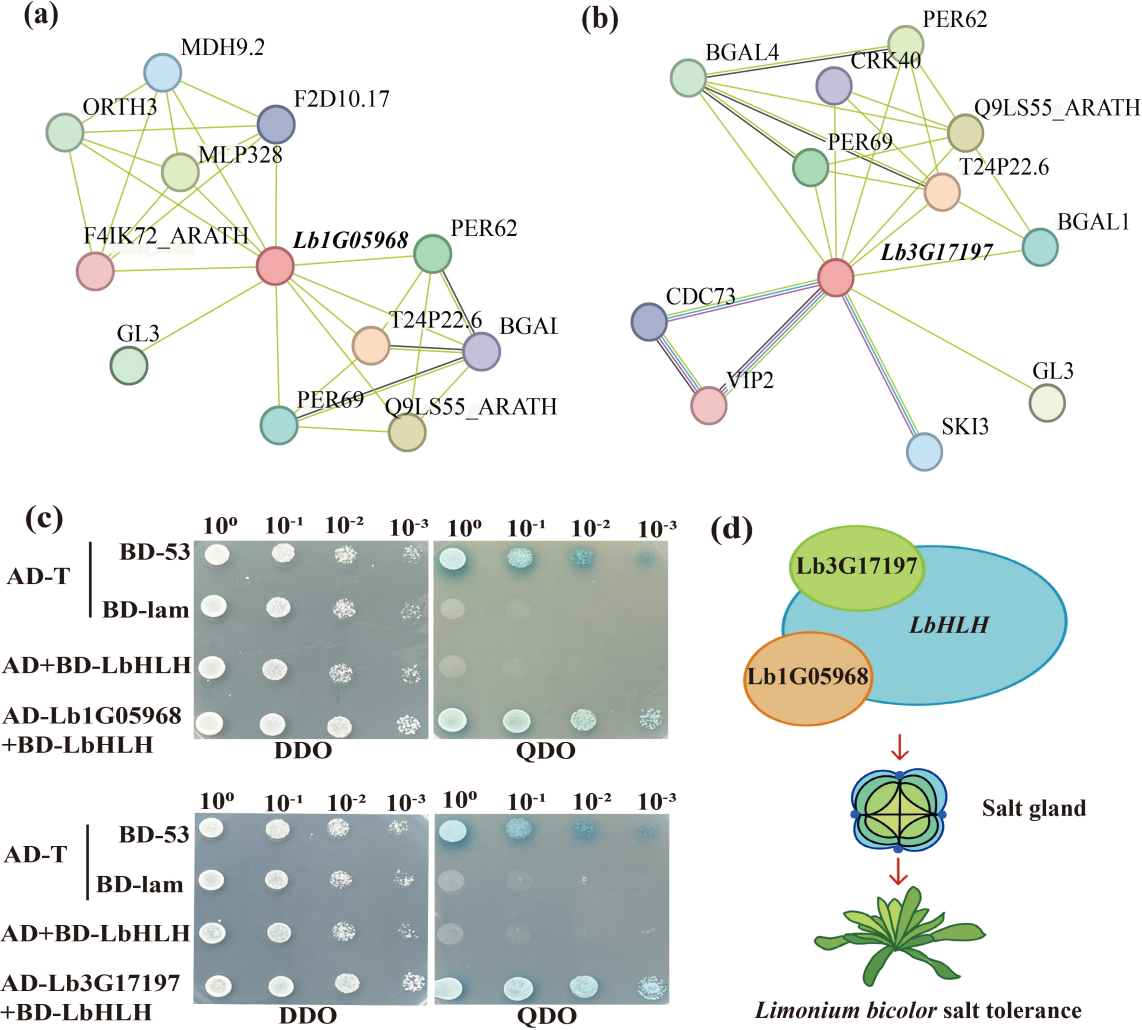
The protein interaction map of *Lb1G05968* and *Lb3G17197* and the yeast interaction, regulatory model with *LbHLH* (Lb1G04899). **(a)** Protein–protein interaction network of Lb1G05968 in Arabidopsis. **(b)** Protein–protein interaction network of Lb3G17197 in Arabidopsis. **(c)** Yeast two-hybrid assay showing that the proteins encoded by Lb1G05968 and Lb3G17197 interact with LbHLH. AD, GAL4 activation domain from the pGADT7 vector; BD, GAL4 DNA-binding domain from the pGADT7 vector. The pGBKT7-*LbHLH* construct was introduced in yeast cells to test the autoactivation of LbHLH. The constructs pGADT7-Lb1G05968 and pGADT7-Lb3G17197 were co-introduced into yeast cells together with pGBKT7-*LbHLH* to test interaction. All transformants were plated on double dropout (DDO) medium (synthetic defined (Ananieva et al.)/−Leu/−Trp). Positive transformants were spotted onto DDO and quadruple dropout (QDO) medium (SD/−Ade/−His/−Leu/−Trp/X-α-gal. **(d)** Schematic diagram of the interaction between *Lb1G05968*, *Lb3G17197* and *LbHLH* in regulating salt gland development.

We utilized the STRING protein interaction database to predict the protein–protein interaction network between the proteins encoded by Lb1G05968 and Lb3G17197 and Arabidopsis proteins (**Figures 5a, b**). This analysis suggested that Lb1G05968 and Lb3G17197 can both potentially interact with GLABRA 3 (GL3), a bHLH-type transcription factor that, together with GL1 and TTG1, regulates trichome development in Arabidopsis (Payne et al., 2000). In *L. bicolor*, the gene with the closest homology to *GL3* is *LbHLH*, which promotes trichome differentiation and enhances plant salinity tolerance (Wang et al., 2021). Previous studies demonstrated that the WD40 protein LbTTG1 interacts with LbHLH to suppress salt gland formation in epidermal structures of *L. bicolor* leaves. To test whether the WD40 family members encoded by Lb1G05968 and Lb3G05971 similarly interact with LbHLH, we conducted yeast two-hybrid assays. Indeed, we determined that the proteins encoded by Lb3G05971 and Lb1G05968 interact with LbHLH. (**Figure 5c**) These results suggest that members of the LbWD40 family may form functional complexes with LbHLH family members to regulate salt gland development in *L. bicolor*. Based on the above experiments, we constructed a regulatory network diagram among these three genes (**Figure 5d**).

## Discussion

In this study, we elucidated the 367 members of the WD40 gene family in *L. bicolor* via a systematic analysis of their coding and genomic sequences, intron–exon structures, and expression patterns during salt gland development and under salt stress. Moreover, we demonstrated a role for two *LbWD40* genes in salt gland development, based on the phenotypes of plants silenced for each gene via VIGS, and that their encoded proteins from subfamily 6 of the LbWD40 family can interact with the bHLH transcription factor LbHLH to positively regulate salt gland development. This research provides a theoretical foundation for elucidating the functional characteristics and regulatory networks of the WD40 protein family, while establishing a preliminary research basis for screening salinity tolerance–related WD40 genes in *L. bicolor*.

### Evolutionary analysis of the *LbWD40* gene family

To explore the evolutionary relationships of WD40 proteins from *L. bicolor*, we reconstructed a phylogenetic tree encompassing members from *L. bicolor* and Arabidopsis. This phylogenetic analysis revealed that closely related members share consistent motif compositions, indicating the high conservation of the WD40 family across plant species. These results suggest that WD40 genes from different plants may have originated from a common ancestor (Olsen et al., 2005; Liu et al., 2023b; Shen et al., 2025). Large-scale expansions of gene family members drive plant adaptive evolution (Hussain et al., 2023). Notably, the model plant Arabidopsis possesses far fewer WD40 genes (213) than *L. bicolor* (367). The number of WD40 genes in tomato (207) (Yan et al., 2023), peach (220) (Feng et al., 2019) is also substantially lower than that in *L. bicolor* (367). This comparison further demonstrates a lineage-specific expansion of WD40 genes in *L. bicolor*, which likely contributes to its exceptional salt tolerance. Moreover, many of the *LbWD40* family genes are highly expressed during early salt gland development and under salt stress conditions. In this study, we confirmed a positive regulatory role for Lb1G05968 and Lb3G17197 in salt gland development through VIGS-mediated silencing. The expansion of the WD40 gene family in *L. bicolor*, potentially driven by adaptation to salt stress, may reflect genomic evolutionary strategies that enhance the environmental adaptability of this species.

### Possible candidate WD40 proteins contributing to salt gland development

The developmental similarity between trichome formation in Arabidopsis and salt gland development in *L. bicolor* has been established (Yuan et al., 2015b). The WD40 protein LbTTG1, homologous to AtTTG1 in Arabidopsis, interacts with LbHLH and negatively regulates salt gland development in *L. bicolor*. By contrast, AtTTG1 promotes trichome development by forming a complex with MYB and bHLH transcription factors, thereby activating downstream target gene expression (Liu et al., 2017a). This divergent regulatory mechanism highlights the functional complexity of the LbWD40 protein family, meriting an in-depth investigation. Importantly, our discovery of two LbWD40 regulators (Lb1G05968 and Lb3G17197) challenges the prevailing single gene regulatory model in epidermal structures (Moog et al., 2022), indicating *L. bicolor* may evolve more complex WD40 networks to determine salt gland development. Consequently, we identified 367 WD40 proteins encoded by *L. bicolor* and identified two additional WD40 proteins in addition to LbTTG1 involved in salt gland development, which also interact with LbHLH. These findings suggest that the LbWD40 family likely harbors numerous proteins with as-yet-undiscovered roles associated with salt gland development, collectively forming a sophisticated regulatory network. The polygenic regulatory mechanism governing salt gland development within the WD40 family warrants systematic studies.

### WD40 proteins of *L. bicolor* are associated with the development of epidermal structures, particularly the formation of salt glands

WD40 proteins play important roles in plant growth and development, as well as in fruit development, acting as scaffolds for the assembly of protein complexes through protein–protein interactions (Stirnimann et al., 2010; Miyata and Nishida, 2011; Li et al., 2012; Chen et al., 2022; Yu et al., 2023). Our results demonstrate that in L. bicolor, WD40 genes show particularly intriguing expression patterns during salt gland development, suggesting their probable involvement in this specialized epidermal structure formation.

The observed high expression of multiple LbWD40 genes during early salt gland differentiation stages may indicate their functional importance in the initiation of salt gland development. This expression pattern bears resemblance to the role of WD40 proteins in other plant secretory structures. For instance, in quinoa, the WD40 proteins REBC (Reduced levels of epidermal bladder cells) and REBC-like1 determine salt bladder development (Moog et al., 2022). Given that salt bladders and salt glands belong to similar salt secretory structures (Yuan et al., 2016). It is plausible that LbWD40 genes may operate through analogous mechanisms to regulate salt gland formation.

The marked responsiveness of LbWD40 genes to salt treatment further supports their potential role in salt gland function. This observation aligns with previous findings in persimmon (*Diospyros virginiana*), where the WD40 protein TTG1 participates in stress-responsive pathways by forming MBW complexes (Gil-Muñoz et al., 2020).

Notably, our transcriptome data revealed stage-specific expression patterns of LbWD40 genes during salt gland development. This temporal regulation might reflect their diverse functions at different developmental phases, possibly including early-stage cell fate determination, intermediate-stage structural differentiation, and mature-stage functional maintenance. Such multifaceted involvement would be consistent with the known pleiotropic effects of WD40 proteins in other systems, like Arabidopsis GTS1 which regulates both seed germination and biomass accumulation (Gachomo et al., 2014).

While our results strongly suggest the involvement of LbWD40 genes in salt gland development, several questions remain unclear. WD40 genes are proposed to be likely operate through integrated transcriptional-translational cascades. In tomato, a WD40 gene’s promoter contains metabolic/developmental cis-elements (O2/RY), while its protein product engages in functional complexes (Yan et al., 2023). LbWD40 genes similarly employ dual regulatory tiers-promoter-driven expression control and protein interaction-mediated effector functions-to orchestrate salt gland development.

The potential manipulation of LbWD40 genes could have significant implications for improving salt tolerance in crops. Given that salt glands represent an evolutionary adaptation to saline environments, enhancing their development or function through WD40 gene regulation might offer a novel strategy for crop improvement. This approach would be particularly valuable in light of increasing soil salinization worldwide.

### Role of LbWD40 proteins in salinity tolerance

WD40 proteins play extensive roles in plant growth and metabolism (Ananieva et al., 2008), as well as in abiotic stress responses (Liu et al., 2017b; Zhi et al., 2022; Eguchi et al., 2024). Our experimental results strongly suggest that WD40 genes in *L. bicolor* likely function as key regulators in salt stress adaptation, particularly through modulating salt gland development and function.

The stress-responsive characteristics of LbWD40 genes appear to be evolutionarily conserved across plant species. In cotton, GhWD40 interacts with ABA signaling to maintain osmotic balance (Hu et al., 2016), while in soybean, GmWD40-NAC complexes activate antioxidant defenses (Yang et al., 2020). Our results extend these findings by revealing a probable connection between WD40 proteins and specialized salt-secreting structures. The reduced salt tolerance in silenced plants particularly suggests that LbWD40s might integrate stress signaling with salt gland development, possibly through mechanisms analogous to the HSP70-WD40 complexes that mitigate heat stress in tomato (Zhu et al., 2022).

The observed phenotypes from VIGS silencing experiments provide compelling evidence for the involvement of LbWD40 genes in salinity tolerance. Silencing Lb1G05968 or Lb3G17197 via VIGS resulted in a lower salt gland density and diminished salinity tolerance compared to controls. Enhanced DAB and NBT staining intensity revealed greater stress-induced damage in plants silenced for Lb1G05968 or Lb3G17197 relative to the control. These findings collectively suggest that LbWD40 proteins may orchestrate salt tolerance through a dual mechanism: coordinating ROS scavenging systems and regulating salt gland formation. This proposed mechanism aligns well with recent reports on WD40 proteins in mangrove salt glands (Song et al., 2024), though the exact molecular pathways in *L. bicolor* remain to be elucidated. The complete interaction networks remain to be fully characterized, and the cis-regulatory elements controlling stress-induced expression of these genes warrant investigation.

The WD40 genes in *L. bicolor* were likely respond to abiotic stress, particularly salt stress. These findings substantiate the potential role of WD40 genes in *L. bicolor* salt acclimation mechanisms. Our results establish a crucial foundation for further investigations into WD40-mediated salinity tolerance strategies in halophytes.

## Materials and methods

### Plant materials and growing conditions

*Limonium bicolor* seeds were collected from plants growing on the saline-alkali soil of Dongying, Shandong Province (37°30′N, 117°86′E). After collection, the seeds were thoroughly dried and stored in conventional low-temperature storage at 4°C until use. Seeds were surface sterilized as follows: First, the seeds were soaked in 75% (v/v) ethanol for 5 min on a shaker (180 rpm), followed by incubation in 6% (v/v) sodium hypochlorite (NaClO) for 19 min. The seeds were then rinsed 4–5 times with sterile distilled water and soaked for 20 min until the seed coat softened and began to detach. The surface-sterilized seeds were transferred to basal Murashige and Skoog (MS) medium solidified with 1% (w/v) agar. Growth conditions were set as follows: light intensity 580 μmol·m−^2^·s−^1^, photoperiod of 17-h light/7-h dark, humidity 68%, and temperature 29°C (day)/22°C (night).

### Identification of the *LbWD40* gene family of *L. bicolor*

WD40 genes were systematically identified using a domain-based mining strategy based on the gene of *Limonium bicolor* (PRJNA753199). Initial screening was performed against the Pfam database (PF00400) using Pfam_scan to detect WD40 domain-containing proteins, followed by SMART (v9.0) (https://smart.embl.de/) validation which confirmed the absence of duplicate genes. Further verification involved diamond BLAST searches (E-value <1e-5) against five major databases (NR, TrEMBL, SwissProt, KEGG, and KOG), with final manual validation conducted via NCBI BLAST. Finally, a list of 367 WD40 proteins was identified as being encoded by the *L. bicolor* genome. ExPASy (https://www.expasy.org/) was used to predict the physical characteristics of these proteins, including isoelectric point (pI), molecular mass (MW), and subcellular localization.

### Phylogenetics, intron–exon structure, and motif composition

After acquiring the complete set of WD40 protein amino acid sequences for both *Arabidopsis and L. bicolor*, we conducted phylogenetic analysis using MEGA7.9, beginning with multiple sequence alignment of WD40 proteins from *Arabidopsis* and *L. bicolor* using the MUSCLE algorithm (employing gap penalties of -400 for opening and 0 for extension, with UPGMB clustering). Subsequently, we constructed a maximum likelihood (ML) tree under the JTT+G model (with gamma shape parameter α=0.5) and assessed nodal support with 1000 bootstrap replicates. The substitution model was selected based on the Bayesian Information Criterion (BIC). The phylogenetic tree was constructed using maximum likelihood method with 1,000 bootstrap replicates. The gene models and intron–exon structures of all *LbWD40* genes were assessed with an online service (http://gsds.cbi.pku.edu.cn/) by comparing the sequences of the cDNA and the genomic sequence. The conserved motifs of WD40 protein candidates were determined by MEME analysis (https://meme-suite.org/tools/meme).

### Analysis of chromosomal positions, gene duplications, and synteny of *LbWD40* genes with homologs in other plants

The mapping information for all *LbWD40* genes was visualized as a Circos plot using Circos v0.69-9 (http://circos.ca/). LbWD40 gene duplication events were assessed using the multicolinear scanning toolkit (MCScanX) with the following parameters: E-value < 1e-10, minimum aligned sequence identity > 75%, and gap size < 25 genes. The syntenic relationship between the *LbWD40* genes and *WD40* genes from *Arabidopsis thaliana*, *Beta vulgaris*, and *Fagopyrum tataricum* were determined using Dual Synteny Plotter software.

### The expression patterns during leaf development and NaCl treatment

Building upon our previously acquired transcriptome datasets (PRJNA752802 and PRJNA752940) of *Limonium bicolor*, we systematically analyzed the expression patterns of identified WD40 family genes across different developmental stages (A-E), as enriched from the first true leaf at each stage, and under salt stress treatments (0, 12, 24, 48, and 72 hours). The raw RNA-seq data underwent quality control filtering using fastp with default parameters, followed by alignment of high-quality reads to the reference genome using HISAT2. Gene expression levels were quantified as FPKM values through the Cufflinks pipeline. For the WD40 gene family specifically, we extracted the FPKM expression matrix, performed row-wise z-score normalization, and conducted heatmap visualization analysis, thereby comprehensively revealing both stage-specific expression characteristics and salt stress response patterns of this gene family.

### Construction of expression vectors

An adequate quantity of first true leaves of *L.* bicolor was collected at various growth stages (A–E). After flash-freezing in liquid nitrogen, samples were thoroughly ground into a fine powder using pre-chilled mortars. Total RNA was extracted using a FastPure Plant Total RNA Isolation Kit (RC411-C1; Vazyme Biotech, Nanjing). Following quality verification, high-quality first-strand cDNA was synthesized according to the protocol provided with the SPARKscript II RT Plus Kit (with gDNA remover; Sparkjade Biotechnology, Shandong). Full-length amplification of target genes was performed using cDNA as template with specific primers (e.g. Lb1G05968-S, Lb1G05968-A)(**Supplementary Table S2**) designed by Primer 5.0 software.

Specific primers (e. g. Lb1G05968-1300-S, Lb1G05968-1300-A) containing homologous arms for the pCAMBIA1300-35S-sGFP vector were also designed to amplify fragments of *LbWD40* genes containing SalI restriction sites. The pCAMBIA1300-35S-sGFP vector empty vector was linearized by digestion with the SalI restriction enzyme, and the *LbWD40* fragments were purified following the manufacturer’s instructions for the Vazyme FastPure Gel DNA Extraction Mini Kit. Following the manufacturer’s instructions for the Vazyme ClonExpress II One Step Cloning Kit, the SalI-digested *LbWD40* amplicons were ligated with the linearized pCAMBIA1300-35S-sGFP vector via homologous recombination, ultimately obtaining the pCAMBIA1300-Lb1G05968 and pCAMBIA1300-Lb3G17197 constructs.

### Subcellular localization

The above plasmids pCAMBIA1300-Lb1G05968 and pCAMBIA1300-Lb3G17197, as well as the empty vector pCAMBIA1300-35S-*sGFP*, were introduced into Agrobacterium (*Agrobacterium tumefaciens*) strain GV3101 competent cells via electroporation. Agrobacterium in 25 mL YEB medium (containing 50 μg/ml kanamycin, 50 μg/ml rifampicin, and 5 μl of 20 μM acetosynringone) overnight at 28°C at 200 rpm. The bacterial cells were then collected by centrifugation at 6000 rpm for 8 min and resuspended in 10 mL infiltration buffer (10 mM MgCl_2_, 10 mM MES, pH 5.6) to an OD_600_ of 0.6, followed by addition of acetosyringone to a final concentration of 150 mM and incubation at 28°C for 2 hours. The bacterial suspension was infiltrated into the abaxial surface of *Nicotiana benthamiana* leaves using a 10 mL disposable syringe until complete saturation was achieved. After infiltration, the plants were kept in darkness for 2-3 days (Goodin et al., 2002).

Subcellular localization observation was performed using a confocal laser scanning microscope. Nuclei were stained with 4',6-diamidino-2-phenylindole (DAPI) and observed under 358 nm excitation wavelength. The plasma membranes were labeled with FM4-64 fluorescent dye and detected at 559 nm excitation wavelength. All fluorescence images were acquired and analyzed using identical parameters to ensure comparability of experimental results (Ng et al., 2010).

### Phenotypic analysis of *LbWD40*-VIGS plants in *L. bicolor*

Specific primers containing EcoRI and KpnI restriction sites were designed using CE Design V1.04 primer design software to amplify specific fragments of the coding sequences of Lb1G05968 and Lb3G17197, using reverse-transcribed cDNA as template. The ELKRNA2 vector was also subjected to a double digestion with KpnI and EcoRI, after which the amplified products were directionally ligated into the linearized ELKRNA2 vector, generating the VIGS plasmids ELKRNA2-Lb1G05968 and ELKRNA2-Lb3G17197 (Zhou et al., 2024).

The resulting plasmids were introduced into Agrobacterium competent cells; positive colonies were used to inoculate liquid YEB medium (containing 50 μg/ml kanamycin, 50 μg/ml rifampicin, and 10 μl of 20 μM acetosynringone) for culture at 28°C at 200 rpm. Bacterial cells were collected by centrifugation at 6000 rpm for 8 min and resuspended in infiltration buffer (10 mM MgCl_2_, 10 mM MES, 100 μM acetosynringone), followed by dark incubation treatment for 2h and equal-volume mixing with an Agrobacterium cell suspension harboring the helper plasmid pRNA1. The cell mixture was introduced into the leaves of six-leaf-stage *L. bicolor* seedlings via infiltration. Following dark culture for 2 days and photoperiod adaptation for 3-4 days of the infiltrated plants, newly emerged leaves were selected for phenotypic observation, and salt secretion was assessed using an improved leaf disk method (Yuan et al., 2018b).

Prior to experiments, residual salts were removed from the leaf surfaces with distilled water. Leaf discs (10 mm in diameter) were prepared with a hole puncher and placed in Petri dishes containing 200 mM NaCl solution with the abaxial side facing downward; the leaf discs were then covered with mineral oil to prevent evaporation. After 24 hours, secreted droplets were collected from the surface of leaf discs, and sodium ion content was quantified using a high-sensitivity flame photometer (flame photometer 410, Sherwood, Britain). After removing the liquid droplets, the leaf discs were fixed with an ethanol-acetic acid mixed solution (3:1, v/v), followed by chlorophyll extraction through rinsing in 70% (v/v) ethanol. Subsequently, the samples were cleared in Hoyer’s solution, and the morphological characteristics of salt glands were observed under a fluorescence microscope equipped with a differential interference contrast (DIC, ECLIPSE 80i, Nikon, Japan) device (Yuan et al., 2013). The number of salt glands was recorded and the sodium ion secretion rate per individual salt gland was calculated as previously described.

### Yeast two-hybrid assays

The full-length coding sequences of Lb1G05968 and Lb3G17197 were individually and directionally inserted into the pGADT7 vector at the NdeI restriction site via homologous recombination (Vazyme ClonExpress II quick cloning kit), yielding the plasmids AD-Lb1G05968 and AD-Lb3G17197. The full-length coding sequence of *LbHLH* was similarly subcloned into the pGBKT7 (BD) vector using the NdeI restriction site. As negative controls or positive controls, the empty vector pGADT7-T was introduced in Y2H Gold cells together with pGBKT7-lam or pGBKT7-53. All plasmids were co-introduced as appropriate pairs into the Y2H Gold yeast strain following the standard protocol of Clontech’s Yeastmaker Yeast Transformation System 2 (Product No. 630439).

Positive transformants were initially screened on synthetic defined (SD) medium lacking leucine and tryptophan (SD/−Leu/−Trp). Positive colonies were subsequently grown in liquid SD/−Leu/−Trp medium and serially diluted before being spotted onto SD/−Leu/−Trp medium and quadruple dropout medium (SD/−Ade/−His/−Leu/−Trp) containing 40 mg/ml X-α-gal. The plates were incubated at 29°C for 3-4 days.

### Statistical analysis

Statistical analyses were performed using SPSS software (v26.0), including Duncan’s multiple range test (α=0.05) and Student’s t-test. Group differences were evaluated through mean comparison and ANOVA with orthogonal contrast analysis.

## Data Availability

The original contributions presented in the study are included in the article/**Supplementary Material**. Further inquiries can be directed to the corresponding authors.

## Supplementary material

Supplementary Figure 1 Gene models of WD40 genes in *Limonium bicolor*. The green boxes represent exons, the red lines represent introns.

Supplementary Figure 2 Motif composition of WD40 family members of *Limonium bicolor*. Different potential functional motifs are shown as colored boxes and were predicted by MEME analysis.

Supplementary Figure 3 Expression analysis of the WD40 gene family of *Limonium bicolor.* Heatmap representation of expression levels for the members of the WD40 gene family. The genes were subjected to hierarchical clustering at specific developmental stages and time points

Supplementary Figure 4 Expression analysis of the WD40 gene family of *Limonium bicolor* under salt treatment. Heatmap representation of expression levels for the members of the WD40 gene family under control and treatment with 200 mM NaCl. The genes were subjected to hierarchical clustering.

Supplementary Figure 5 The amino acid sequence alignment diagram of Lb1G05968 and Lb3G17197 compared with REBC from *Chenopodium quinoa*.
