# Supplementary figures and images for: The WD40 gene family in recretohalophyte Limonium bicolor: genomic identification and functional analysis in salt gland development and salinity tolerance
Source: Front Plant Sci. 2025 Jul 30;16:1629604. doi: 10.3389/fpls.2025.1629604 (PMC12343514; doi:10.3389/fpls.2025.1629604)

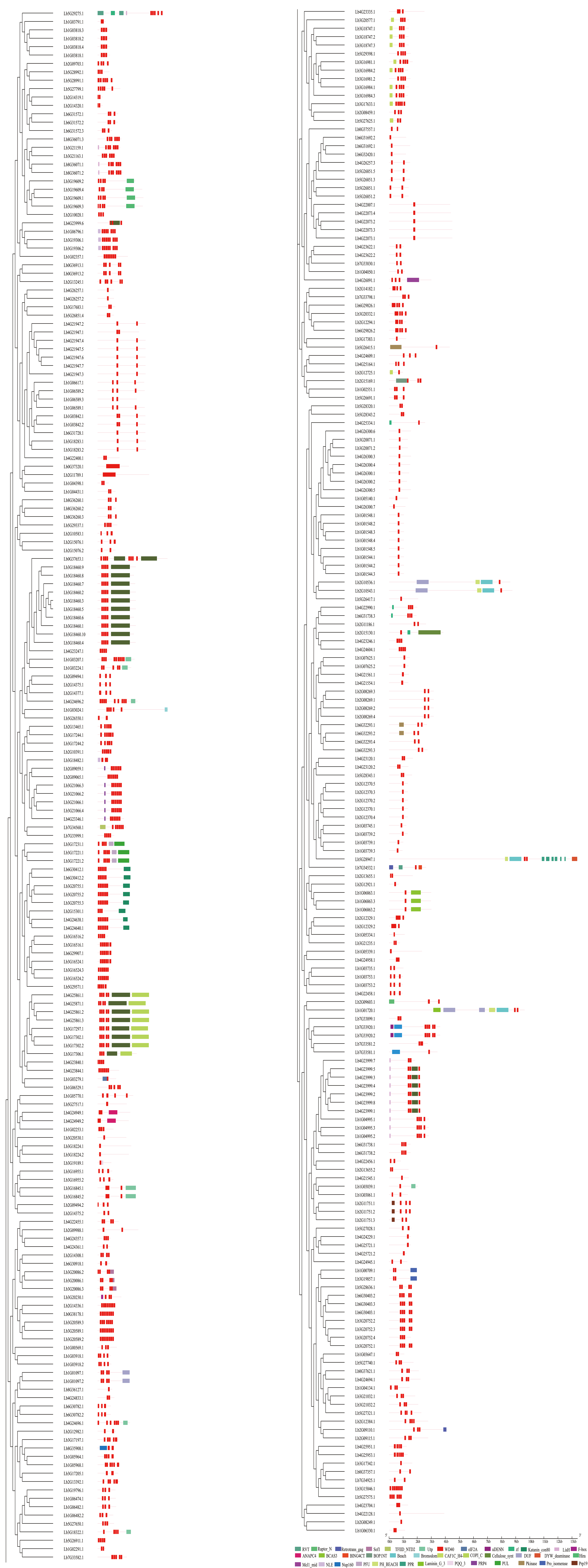

Supplement: Supplementary Figure 2 — Motif composition of WD40 family members of Limonium bicolor. Different potential functional motifs are shown as colored boxes and were predicted by MEME analysis. [file Image2.jpeg]

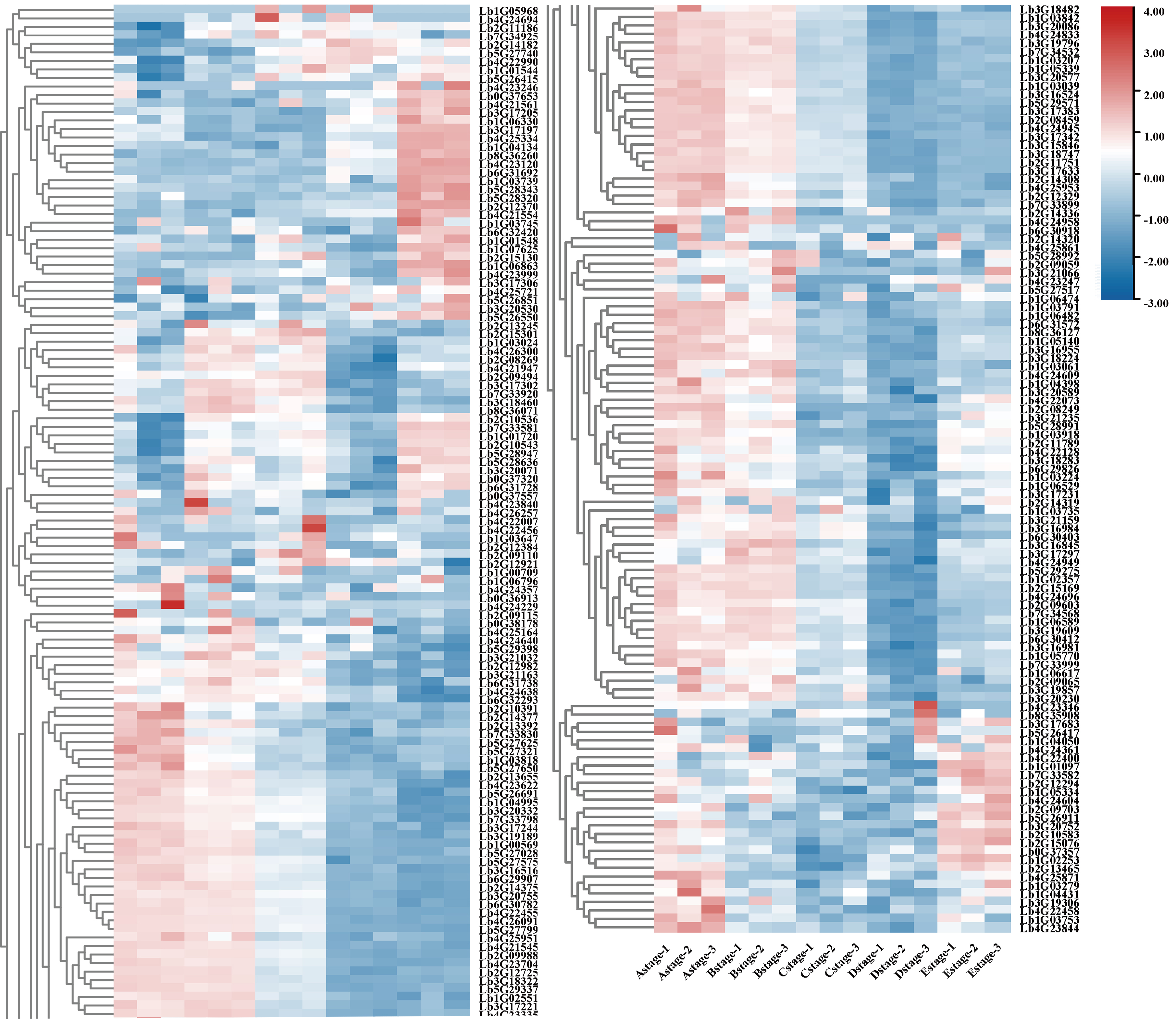

Supplement: Supplementary Figure 3 — Expression analysis of the WD40 gene family of Limonium bicolor. Heatmap representation of expression levels for the members of the WD40 gene family. The genes were subjected to hierarchical clustering at specific developmental stages and time points [file Image3.jpeg]

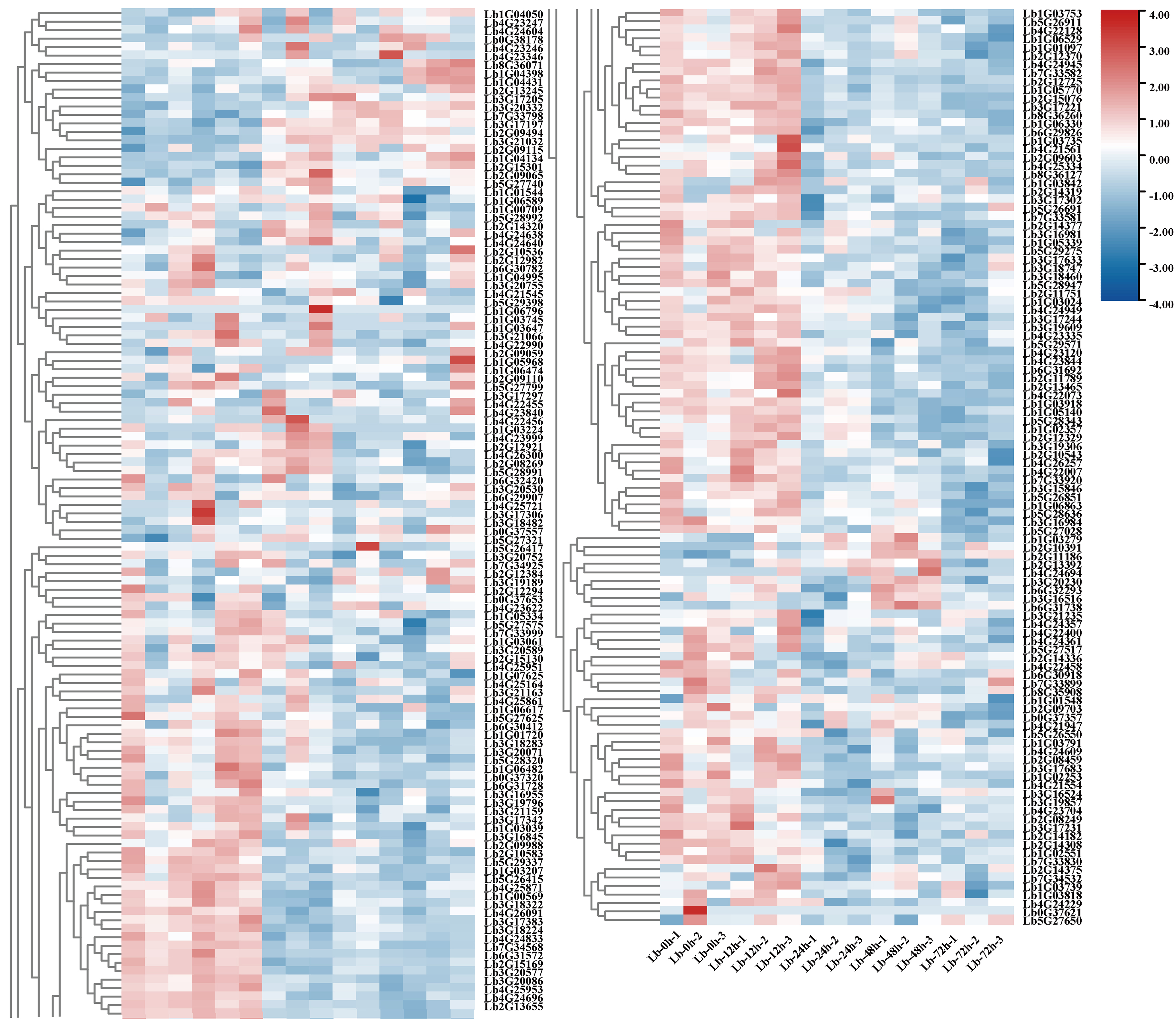

Supplement: Supplementary Figure 4 — Expression analysis of the WD40 gene family of Limonium bicolor under salt treatment. Heatmap representation of expression levels for the members of the WD40 gene family under control and treatment with 200 mM NaCl. The genes were subjected to hierarchical clustering. [file Image4.jpeg]

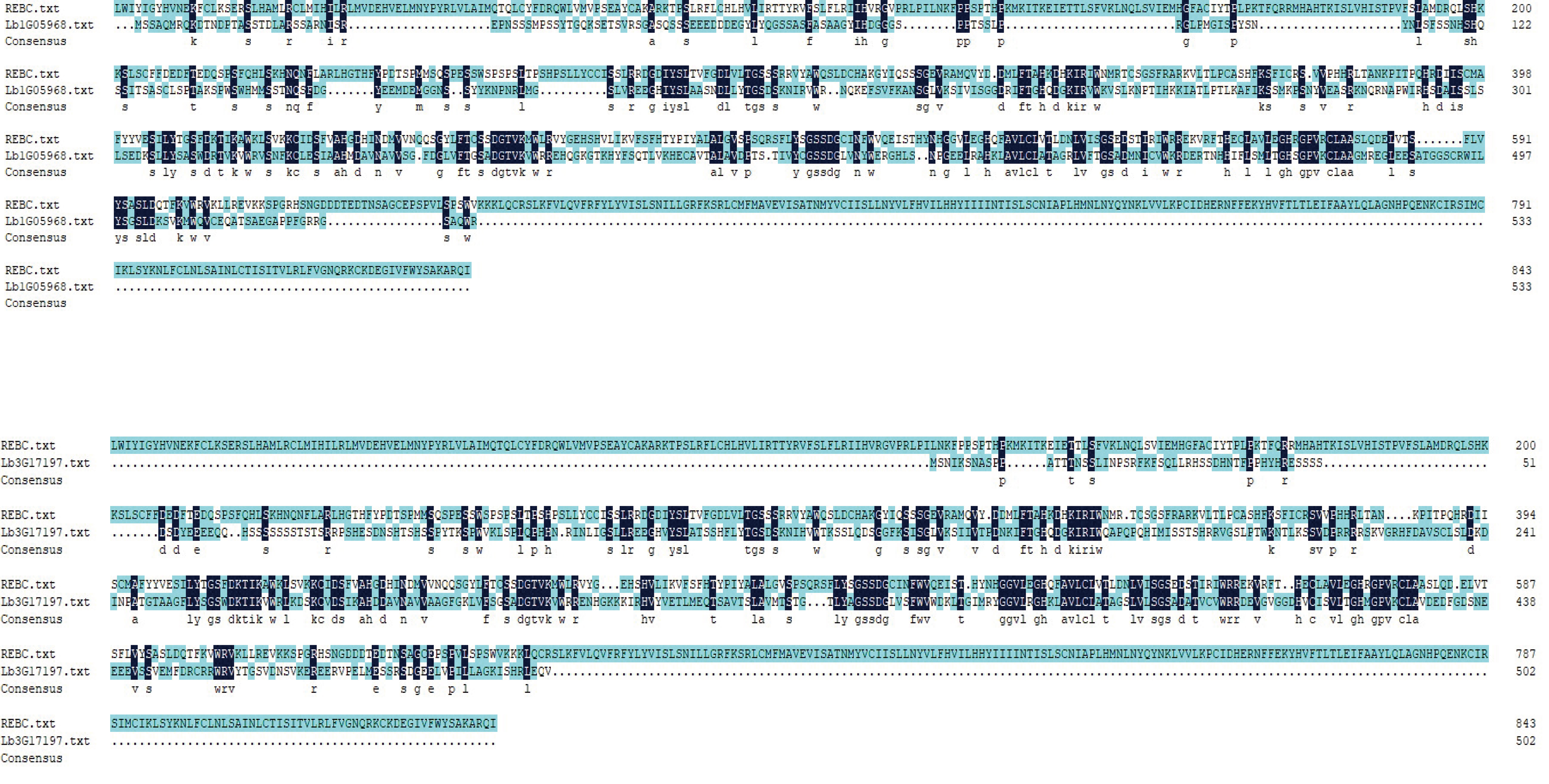

Supplement: Supplementary Figure 5 — The amino acid sequence alignment diagram of Lb1G05968 and Lb3G17197 compared with REBC from Chenopodium quinoa. [file Image5.jpeg]
